# Perioperative antithrombotic management in degenerative spine surgery: a multicenter cohort of 767 consecutive patients

**DOI:** 10.3389/fmed.2026.1831400

**Published:** 2026-08-26

**Authors:** Giuseppe Corazzelli, Sergio Corvino, Davide De Los Rios, Diego Gentile, Chiara Di Domenico, Francesco Corazzelli, Gennaro De Rosa, Francesco Fiore, Cristiana Germano, Marco Filippelli, Niccolò Colella, Francesco Ricciardi, Alessandro D’Elia, Settimio Leonetti, Maria Sacco, Vincenzo Meglio, Valentina Pizzuti, Paolo Di Russo, Nicola Gorgoglione, Giandomenico Petrella, Ciro Mastantuoni, Maria Rosaria Scala, Michelangelo de Angelis, Alberto De Bellis, Valentina Cioffi, Fulvio Aloj, Luigi Sigona, Antonio Bocchetti, Salvatore Di Colandrea, Giuseppe Coscia, Maria Beatrice Passavanti, Alessandra Cuomo, Pasqualino De Marinis, Carlo Gabriele Tocchetti, Oreste de Divitiis, Mauro Mormile, Plinio Cirillo, Giuseppe Catapano, Raffaele de Falco, Sergio Paolini, Vincenzo Esposito, Gualtiero Innocenzi

**Affiliations:** 1 Department of Neurosurgery, Santa Maria delle Grazie Hospital, ASL Napoli 2 Nord, Pozzuoli, Italy; 2 Department of Human Neurosciences, Sapienza University of Rome, Rome, Italy; 3 Department of Neurosurgery, Presidio Ospedaliero “Ospedale del Mare”, Naples, Italy; 4 University of Naples Federico II, Naples, Italy; 5 Department of Women, Child, General and Specialistic Surgery, University of Campania “L. Vanvitelli”, Naples, Italy; 6 Department of Neurosciences and Reproductive and Odontostomatological Sciences, Neurosurgical Clinic, University of Naples Federico II, Naples, Italy; 7 Department of Advanced Medical and Surgical Sciences, University of Campania “Luigi Vanvitelli”, Naples, Italy; 8 Department of Translational Medical Sciences, University of Naples Federico II, Naples, Italy; 9 Department of Advanced Biomedical Sciences, Università degli Studi di Napoli “Federico II”, Napoli, Italy; 10 Department of Maxillofacial Surgery, Azienda Ospedaliera “San Pio”, Benevento, Italy; 11 Department of Clinical Medicine and Surgery, University of Naples Federico II, Naples, Italy; 12 Department of Neurosurgery, IRCCS Neuromed, Pozzilli, Italy; 13 Department of Anaesthesiology and Intensive Care Medicine, Santa Maria delle Grazie Hospital, ASL Napoli 2 Nord, Pozzuoli, Italy; 14 Department of Neurosurgery, Azienda Ospedaliera di Rilievo Nazionale “Sant’Anna e San Sebastiano”, Caserta, Italy; 15 Department of Anesthesiology, IRCCS Neuromed, Pozzilli, Italy

**Keywords:** anticoagulation, antithrombotic therapy, degenerative spine disease, low-dose aspirin, low-molecular-weight heparin, perioperative management, spine surgery

## Abstract

**Background:**

Perioperative management of antithrombotic therapy in degenerative spine surgery remains a challenging clinical issue, particularly in elderly patients with cardiovascular comorbidities requiring chronic anticoagulant or antiplatelet treatment. Although several guidelines address perioperative anticoagulation in high–bleeding-risk procedures, procedure-specific evidence in elective spinal surgery remains limited, and clinical practice is still heterogeneous.

**Methods:**

A multicenter retrospective cohort study was conducted including 767 consecutive patients aged 60 years or older who underwent elective surgery for degenerative spine disease. Surgical procedures included anterior cervical discectomy and fusion, lumbar microdiscectomy for disc herniation, decompression for lumbar spinal stenosis, and monosegmental posterior lumbar interbody fusion. Patients were stratified according to perioperative antithrombotic management into three groups: no chronic antithrombotic therapy, perioperative low-molecular-weight heparin substitution, and temporary perioperative substitution with low-dose aspirin. Intraoperative hemoglobin loss, operative time, postoperative length of stay, and perioperative complications were compared across groups.

**Results:**

Perioperative outcomes were similar among the three antithrombotic management groups across all procedures. Intraoperative hemoglobin loss, operative duration, and postoperative hospitalization showed no significant differences between patients undergoing low-molecular-weight heparin substitution or aspirin continuation and those without chronic antithrombotic therapy. No cases of postoperative spinal epidural hematoma requiring evacuation, myocardial infarction, stroke, deep venous thrombosis, or pulmonary embolism were observed. The observed between-group differences in hemoglobin loss, operative time, and postoperative hospitalization remained within the predefined clinically meaningful margins.

**Conclusion:**

In this multicenter cohort of 767 consecutive patients undergoing common degenerative spine procedures, perioperative antithrombotic management based on low-molecular-weight heparin substitution or low-dose aspirin substitution was not associated with clinically relevant worsening of perioperative outcomes when compared with patients not receiving chronic antithrombotic therapy. These findings suggest that structured perioperative antithrombotic protocols may represent a reasonable strategy in elective degenerative spine surgery.

## 1 Introduction

Degenerative spine disease represents one of the most frequent indications for elective spinal surgery in the elderly population, a demographic in which cardiovascular and cerebrovascular comorbidities requiring chronic antithrombotic therapy are increasingly prevalent (1, 2). The coexistence of degenerative spinal pathology and long-term anticoagulant or antiplatelet treatment poses a major perioperative challenge: surgeons must minimize hemorrhagic risk while avoiding potentially harmful interruptions of thromboprophylaxis (3). This balance is particularly delicate in spine surgery, where postoperative spinal epidural hematoma, although uncommon, may result in severe neurological compromise (4).

Despite the clinical relevance of this issue, the existing literature provides only limited guidance for procedure-specific management of antithrombotic therapy in elective degenerative spine surgery. Although several guidelines and consensus statements provide general recommendations for perioperative antithrombotic management, they offer limited procedure-specific guidance for elective degenerative spine surgery (5–7).

Clinical practice therefore remains heterogeneous. Large surveys, including the AO Spine Anticoagulation Global Survey (8), have demonstrated substantial variation in perioperative antithrombotic strategies across centers worldwide. The available spine-specific evidence is derived mostly from small, single-center series focusing on individual procedure types. Studies evaluating perioperative continuation of low-dose aspirin suggest that aspirin may be continued safely during lumbar decompression (9), yet these findings remain limited to select cohorts (10–12). Even more scarce are data addressing the performance of perioperative low-molecular-weight heparin (LMWH) substitution for patients receiving chronic anticoagulation, where evidence is fragmented and based largely on institutional experience (13–16).

In this context, the absence of unified, multicenter, protocol-driven data represents a critical gap. A comprehensive evaluation of perioperative antithrombotic management across multiple degenerative spine procedures—cervical and lumbar, decompressive and fusion—has the potential to clarify the safety of LMWH substitution and low-dose aspirin continuation within a standardized framework.

To address this gap, we conducted a multicenter retrospective cohort study including 767 consecutive elderly patients undergoing anterior cervical discectomy and fusion (ACDF) (17), lumbar disc herniation microdiscectomy (LMD) (18), lumbar spinal stenosis (LSS) decompression by bilateral laminectomy (LBL) (19–21), or single-level L4–L5 posterior lumbar interbody fusion (PLIF) (1, 22, 23). All patients were managed according to a unified perioperative antithrombotic protocol, allowing direct comparison between those without chronic antithrombotic therapy, those undergoing perioperative LMWH substitution, and those continuing low-dose aspirin. The primary objective of this study was to assess the intraoperative and postoperative safety profile of these strategies and to evaluate whether LMWH substitution and aspirin continuation were associated with clinically meaningful differences in perioperative outcomes compared with patients not receiving chronic antithrombotic therapy.

## 2 Methods

### 2.1 Study setting

This retrospective observational study examined a consecutive cohort of patients who underwent elective surgery for degenerative spinal disease, from January 2022 to December 2024. Clinical, radiological, and surgical information was retrieved from the institutional electronic medical record system and entered into a dedicated database created using Microsoft Excel (Version 16.85, Microsoft Corporation, Redmond, WA, USA). All data were anonymized before analysis, and written informed consent for the use of clinical information had been obtained from all patients at the time of hospitalization, in accordance with institutional and national regulations. This investigation was conducted as a multicenter collaborative study involving four tertiary referral centers, all of which adopted shared patient selection criteria, perioperative management protocols, and follow-up schedules to ensure methodological consistency across participating institutions. According to the applicable national regulations governing retrospective observational studies based exclusively on anonymized clinical data and without any modification of routine clinical management, formal Institutional Review Board approval was not required. The study was conducted in accordance with the ethical principles of the Declaration of Helsinki and its later amendments.

### 2.2 Enrollment criteria

**Figure 1:**
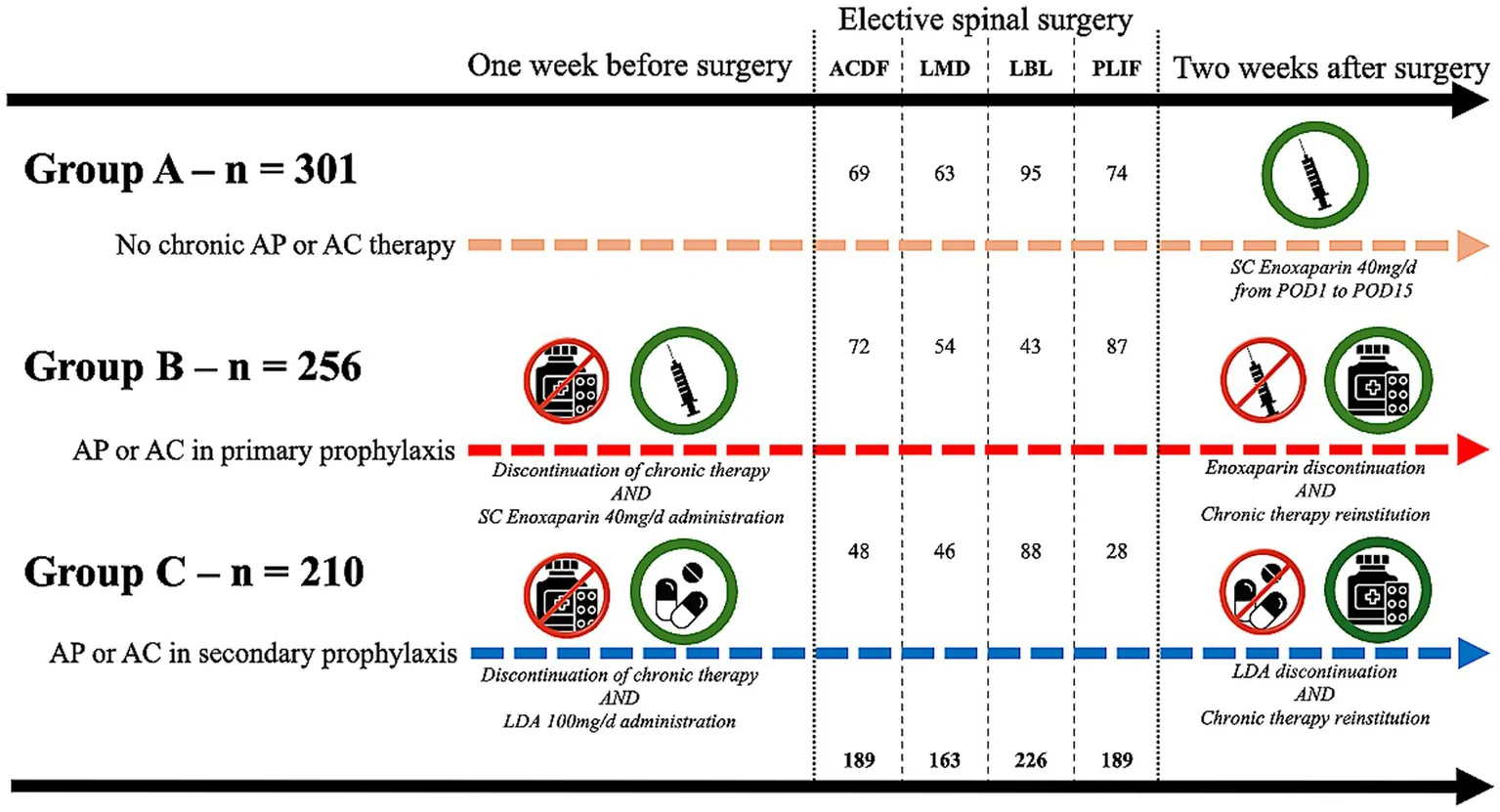
Perioperative antithrombotic management protocol applied to the study cohort. Patients were stratified into three groups according to their chronic antithrombotic therapy. Group A included patients without chronic antiplatelet or anticoagulant therapy who received postoperative low-molecular-weight heparin prophylaxis. Group B included patients receiving antiplatelet or anticoagulant therapy for primary prevention whose therapy was discontinued 7 days before surgery and temporarily replaced with low-molecular-weight heparin. Group C included patients in secondary prevention whose chronic therapy was suspended 7 days before surgery and replaced with low-dose aspirin until postoperative day 15, after which the habitual regimen was resumed. The central panel reports the distribution of surgical procedures within the cohort.

Patients were eligible if they were sixty years of age or older and had a clear clinical and radiological diagnosis of cervical or lumbosacral degenerative pathology. Surgical indications included symptomatic anterior cervical disc disease requiring ACDF (17, 24, 25), lumbar disc herniation (LDH) with radiculopathy or neurological deficit requiring LMD (18), LSS requiring LBL, or low-grade degenerative lumbar spondylolisthesis LSL at L4–L5 requiring PLIF (Figure 1). All patients had undergone an adequate trial of conservative therapy of at least 6 weeks and exhibited persistent symptoms with functional impairment. Radiological assessment followed standard criteria: LSS severity was graded according to Schizas morphology (26, 27), LDH according to the Michigan State University (MSU) classification (21), and LSL according to Meyerding grade I or II with dynamic instability defined by a minimum range-of-movement of 0.5 mm on flexion–extension radiographs (23). Eligibility further required the availability of complete preoperative, perioperative, and three-month postoperative clinical and radiological follow-up, including all outcome measures relevant to the corresponding surgical procedure. Each patient contributed only one index procedure to the study cohort, revision spinal procedures and duplicate patient entries were not eligible for inclusion.

Exclusion criteria included previous spinal surgery, spinal deformities requiring corrective fusion, significant scoliosis, spinal infection, neoplastic or traumatic pathology, incomplete clinical documentation, known bleeding disorders, and chronic antithrombotic regimens incompatible with perioperative substitution. Specifically, patients receiving vitamin K antagonists (VKA) were excluded because their perioperative management is based on INR-guided monitoring and established reversal protocols, similarly dual antiplatelet therapy was excluded owing to its distinct perioperative management strategy, which fell outside the scope of the present study. Patients whose symptoms were inconsistent with radiological findings were also excluded.

### 2.3 Patients’ cohorts

Patients were stratified into three groups according to their habitual antithrombotic therapy. Individuals not receiving antiplatelet or anticoagulant agents formed the first group (Group A). The second group consisted of those in primary cardiovascular prevention (Group B), and the third comprised patients receiving antithrombotic therapy for secondary prevention following Major Adverse CardioVascular Events (MACEs) or coronary interventions (Group C). The term primary prevention referred to chronic antithrombotic therapy prescribed in the absence of previous MACE or thromboembolic events, whereas the term secondary prevention referred to chronic antithrombotic therapy prescribed following a documented MACE, according to the indication of the treating physician. Eligible chronic antithrombotic regimens included single-agent antiplatelet therapy, comprising low-dose aspirin (acetylsalicylic acid) and P2Y12 inhibitors (clopidogrel, prasugrel, or ticagrelor), as well as anticoagulant therapy, comprising direct oral anticoagulants (dabigatran, apixaban, rivaroxaban, or edoxaban), low-molecular-weight heparins (e.g., enoxaparin, nadroparin, or dalteparin), unfractionated heparin, and fondaparinux.

Perioperative management followed a standardized institutional protocol. Patients in primary prevention discontinued their chronic agent approximately 7 days before surgery and received perioperative LMWH, which was continued postoperatively until the habitual therapy was reinstated. Patients in secondary prevention suspended their chronic regimen 1 week before surgery and were transitioned to low-dose aspirin (LDA) throughout the perioperative period. Reinstitution of habitual therapy generally occurred around the fifteenth postoperative day (POD15); however, this interval served as an orientative reference rather than a fixed threshold, and minor adjustments were made when individual cardiovascular risk warranted closer modulation. Patients without prior antithrombotic therapy received postoperative LMWH for venous thromboembolism prophylaxis. The same pharmacological strategy was consistently applied across cervical and lumbar procedures.

### 2.4 Surgical procedures

All operations were performed using standard neurosurgical techniques. ACDF was carried out through a right-sided transverse cervical approach, with discectomy and decompression of neural elements (17, 28–31). Interbody fusion was achieved exclusively using titanium cages, without the addition of grafts or anterior plating, in line with institutional practice. LMD was performed through a midline approach with limited laminotomy and excision of the herniated fragment (32, 33). LSS decompression consisted of bilateral laminectomy, flavectomy, and enlargement of the lateral recesses (19). PLIF included decompression, preparation of the intervertebral space, titanium cage insertion, and bilateral pedicle screw fixation (22, 23, 34). Hemostasis was obtained with bipolar coagulation and adjunctive hemostatic agents when necessary. A closed-suction drain was placed in all LSS and PLIF procedures and was typically removed within the first twenty-four hours.

### 2.5 Variables analyzed

Clinical variables included demographic characteristics, Charlson Comorbidity Index (CCI) (35), preoperative coagulation parameters, operative time, intraoperative hemoglobin loss, drain output, and postoperative length of stay (1). Hemoglobin loss was defined as the absolute difference between the last preoperative hemoglobin value and the first postoperative hemoglobin value obtained on postoperative day 1. Drain output was defined as the cumulative drainage volume recorded from drain placement until drain removal. Pain and disability outcomes, when pertinent to the underlying pathology, were assessed using the Visual Analogue Scale (VAS) and the Oswestry Disability Index (ODI) (36) before surgery and at a three-month follow-up. Postoperative complications were documented for up to 3 months and were classified according to the Clavien–Dindo system (37). Any neurological worsening, reoperation, symptomatic hematoma, transfusion requirement, thromboembolic event, or cardiovascular complication was systematically recorded throughout the postoperative follow-up. Thromboembolic events were identified according to routine clinical practice and investigated by appropriate imaging studies only in the presence of clinical suspicion; no routine imaging surveillance for asymptomatic deep vein thrombosis or pulmonary embolism was performed. In cases of suspected postoperative hematoma, urgent computed tomography or magnetic resonance imaging was performed (38).

### 2.6 Statistical analysis

Statistical analysis was conducted using nonparametric tests because most variables did not follow a normal distribution. Continuous variables were compared using the Mann–Whitney or Kruskal–Wallis test, and categorical variables were analyzed using the chi-square test. Overall comparisons were initially performed across the three study groups. When appropriate, predefined pairwise analyses were subsequently performed between the reference cohort (Group A, patients not receiving chronic antithrombotic therapy) and Group B, as well as between Group A and Group C, using the Mann–Whitney U test.

Clinically meaningful thresholds were predefined as 1 g/dL for hemoglobin loss, 45 min for operative time, and 1 day for postoperative hospitalization, reflecting clinically meaningful differences in perioperative outcomes and were used to contextualize the magnitude of between-group differences (32, 38, 39). These thresholds were defined *a priori* based on clinical judgment and perioperative variability reported in previous spine surgery studies (34, 40, 41). Statistical significance was set at a two-tailed *p* value below 0.05. All analyses were performed using GraphPad Prism (Version 10.2.2, GraphPad Software, Boston, MA, USA). Given the retrospective observational design of the study, no a priori sample size calculation was performed. Instead, all consecutive eligible patients treated during the predefined study period were included in the analysis.

## 3 Results

### 3.1 Baseline characteristics

The unified cohort included 767 consecutive patients aged 60 years or older who underwent ACDF, LDH microdiscectomy, LSS decompression, or monosegmental L4–L5 PLIF. Age was comparable among antithrombotic groups within each surgical category, with mean values of 69.3 years (range 65–78) in ACDF, 66.4 years (range 60–79) in LDH, 68.2 years (range 60–82) in LSS, and 67.8 years (range 60–80) in LSL. Sex distribution remained balanced across procedures, with men representing 66% of ACDF patients, 55% of LDH patients, and approximately 43% of both LSS and LSL cohorts. CCI values were comparable among Groups A, B, and C across all four surgical categories, with no statistically significant between-group differences (all *p* > 0.05). Baseline INR values, functional scores, and radiological severity grading were similarly distributed among Groups A, B, and C in all four surgical categories. Baseline sample’s characteristics are summarized in Table 1.

**Table 1:** Baseline epidemiological and preoperative characteristics of patients undergoing elective spinal surgery according to the perioperative antithrombotic management strategy and surgical procedure.

ACDF	All patients	Group A*	Group B^†^	Group C^‡^	Statistics
No. of patients	189	69 (37)	72 (38)	48 (25)	
Age, yrs	69.25 (3.77)	69.60 (4.06)	70.87 (4.73)	69.79 (4.17)	*p* = 0.26
65–70	117 (62)	51 (74)	43 (60)	23 (48)	
71–75	47 (25)	12 (17)	24 (33)	11 (23)	
>75	25 (13)	6 (9)	5 (7)	14 (30)	
Sex					
Male	125 (66)	48 (70)	43 (60)	34 (71)	*p* = 0.34
Female	64 (34)	21 (30)	29 (40)	14 (29)	
Drug classes					
Low-dose aspirin		—	29 (40)	26 (54)	
P2Y12 inhibitors		—	4 (6)	10 (21)	
DOACs		—	35 (49)	10 (21)	
Heparins		—	4 (6)	2 (4)	
CCI Δ	2.1 (±0.5)	1.9 (±0.8)	2.2 (±0.6)	2.3 (±0.6)	*p* = 0.17
Preoperative neurology					
Myelopathy	147 (78)	48 (70)	58 (80)	41 (85)	*p* = 0.09
Radiculopathy	42 (22)	21 (30)	14 (20)	7 (15)	
Number of levels					
1	87 (46)	31 (45)	34 (47)	22 (46)	*p* = 0.73
2	84 (44)	28 (41)	31 (43)	25 (44)	
3	18 (10)	10 (14)	7 (10)	1 (2)	
Level involved					
C3–C4	62 (20)	21 (20)	17 (15)	24 (29)	*p* = 0.19
C4–C5	93 (30)	28 (26)	39 (34)	26 (31)	
C5–C6	108 (35)	36 (33)	46 (39)	26 (31)	
C6–C7	28 (13)	19 (18)	13 (11)	6 (8)	
C7–T1	4 (2)	3 (3)	1 (1)	0	

LMD	All patients	Group A*	Group B^†^	Group C^‡^	Statistics
No. of patients	163	63 (39)	54 (33)	46 (28)	
Age, yrs	66.67 (4.58)	64.82 (3.92)	67.60 (4.74)	67.23 (4.51)	*p* = 0.80
60–65	83 (51)	27 (43)	23 (42)	33 (72)	
66–70	32 (20)	16 (25)	10 (19)	6 (13)	
>70	48 (29)	20 (32)	21 (39)	7 (15)	
Sex					
Male	89 (55)	34 (54)	28 (52)	27 (59)	*p* = 0.20
Female	74 (45)	29 (46)	26 (48)	19 (41)	
Drug classes					
Low-dose aspirin		—	18 (33)	27 (59)	
P2Y12 inhibitors		—	4 (8)	8 (17)	
DOACs		—	30 (56)	10 (22)	
Heparins		—	2 (3)	1 (2)	
CCI Δ	2.3 (±0.6)	2.1 (±0.5)	2.3 (±0.5)	2.5 (±0.6)	*p* = 0.21
Level involved					
L1–L2	3 (2)	2 (3)	1 (2)	0 (0)	*p* = 0.88
L2–L3	10 (6)	3 (5)	4 (7)	3 (6)	
L3–L4	20 (12)	10 (17)	6 (11)	4 (8)	
L4–L5	92 (57)	33 (52)	33 (61)	26 (56)	
L5–S1	38 (23)	15 (23)	10 (19)	13 (28)	
Site of hernia					
Median	9 (5)	2 (4)	3 (5)	4 (9)	*p* = 0.99
Paramedian left/right	121 (74)	50 (79)	39 (72)	32 (69)	
Intraforaminal	33 (20)	11 (17)	12 (23)	10 (22)	
Predominant side					
Median	9 (5)	2 (3)	3 (5)	4 (9)	*p* = 0.82
Left	72 (44)	31 (49)	23 (43)	18 (39)	
Right	82 (51)	30 (48)	28 (52)	24 (52)	
MSU grade					
1	15 (9)	4 (6)	8 (15)	3 (6)	*p* = 0.67
2	98 (60)	34 (54)	35 (65)	29 (63)	
3	50 (31)	25 (40)	11 (20)	14 (31)	
Ejected hernias	49 (30)	21 (33)	16 (29)	12 (26)	*p* = 0.66
Calcific hernias	26 (16)	13 (20)	9 (16)	4 (9)	*p* = 0.66

LBL	All patients	Group A*	Group B^†^	Group C^‡^	Statistics
No. of patients	226	95 (42)	43 (19)	88 (39)	
Age, yrs	72.16 (4.04)	71.95 (3.91)	72.19 (4.17)	72.38 (4.12)	*p* = 0.77
66–70	99 (44)	43 (45)	19 (44)	37 (42)	
71–75	66 (29)	29 (31)	12 (28)	25 (28)	
>75	61 (27)	23 (24)	12 (28)	26 (30)	
Sex					
Male	134 (59)	58 (61)	25 (58)	51 (58)	*p* = 0.33
Female	92 (41)	37 (39)	18 (42)	37 (42)	
Drug classes					
Low-dose aspirin		—	13 (30)	52 (59)	
P2Y12 inhibitors		—	2 (5)	16 (18)	
DOACs		—	26 (60)	18 (20)	
Heparins		—	2 (5)	2 (2)	
CCI Δ	2.4 (±0.5)	2.2 (±0.7)	2.4 (±0.6)	2.5 (±0.6)	*p* = 0.33
Levels of laminectomy					
1	133 (59)	60 (63)	28 (65)	45 (51)	
2	65 (29)	22 (23)	10 (23)	33 (38)	*p* = 0.40
3	28 (12)	13 (14)	5 (12)	10 (11)	

L4-L5 PLIF	All patients	Group A*	Group B^†^	Group C^‡^	Statistics
No. of patients	189	74 (39)	87 (46)	28 (15)	
Age, yrs	68.36 (5.60)	67.23 (5.15)	68.61 (5.75)	70.55 (5.71)	*p* = 0.06
60–65	75 (40)	34 (46)	34 (39)	7 (25)	
66–70	43 (23)	20 (27)	18 (21)	5 (18)	
71–75	46 (24)	14 (19)	22 (25)	10 (36)	
>75	25 (13)	6 (8)	13 (15)	6 (21)	
Sex					
Male	81 (43)	24 (32)	40 (46)	17 (61)	*p* = 0.25
Female	108 (57)	50 (68)	47 (54)	11 (39)	
Drug classes					
Low-dose aspirin		—	20 (23)	16 (57)	
P2Y12 inhibitors		—	12 (14)	5 (18)	
DOACs		—	50 (57)	6 (21)	
Heparins		—	5 (6)	1 (4)	
CCI Δ	2.5 (±0.6)	2.3 (±0.5)	2.5 (±0.6)	2.7 (±0.7)	*p* = 0.15
Meyerding grade					*p* > 0.5
1	125 (66)	49 (66)	57 (66)	19 (68)	
2	64 (34)	25 (34)	30 (35)	9 (32)	

Values are reported as number (%) or mean ± standard deviation. Continuous variables were compared using the Kruskal–Wallis test, whereas categorical variables were analyzed using the Chi-square test.

ACDF, anterior cervical discectomy and fusion; CCI, Charlson Comorbidity Index; DOACs, direct oral anticoagulants; LMD, lumbar microdiscectomy; LBL, bilateral lumbar laminectomy for spinal stenosis; PLIF, posterior lumbar interbody fusion; MSU, Michigan State University lumbar disc herniation classification; AP, antiplatelet; AC, anticoagulant; LMWH, low-molecular-weight heparin.

Δ: continuous variables were reported as mean ± standard deviation (SD).

*Group A: patients not receiving chronic antiplatelet (AP) or anticoagulant (AC) therapy.

^†^Group B: patients receiving AP or AC therapy for primary prophylaxis who underwent perioperative discontinuation and substitution with low-molecular-weight heparin.

^‡^Group C: patients receiving AP or AC therapy for secondary prophylaxis who underwent temporary discontinuation and perioperative management with low-dose aspirin.

### 3.2 Intraoperative outcomes

**Figure 2:**
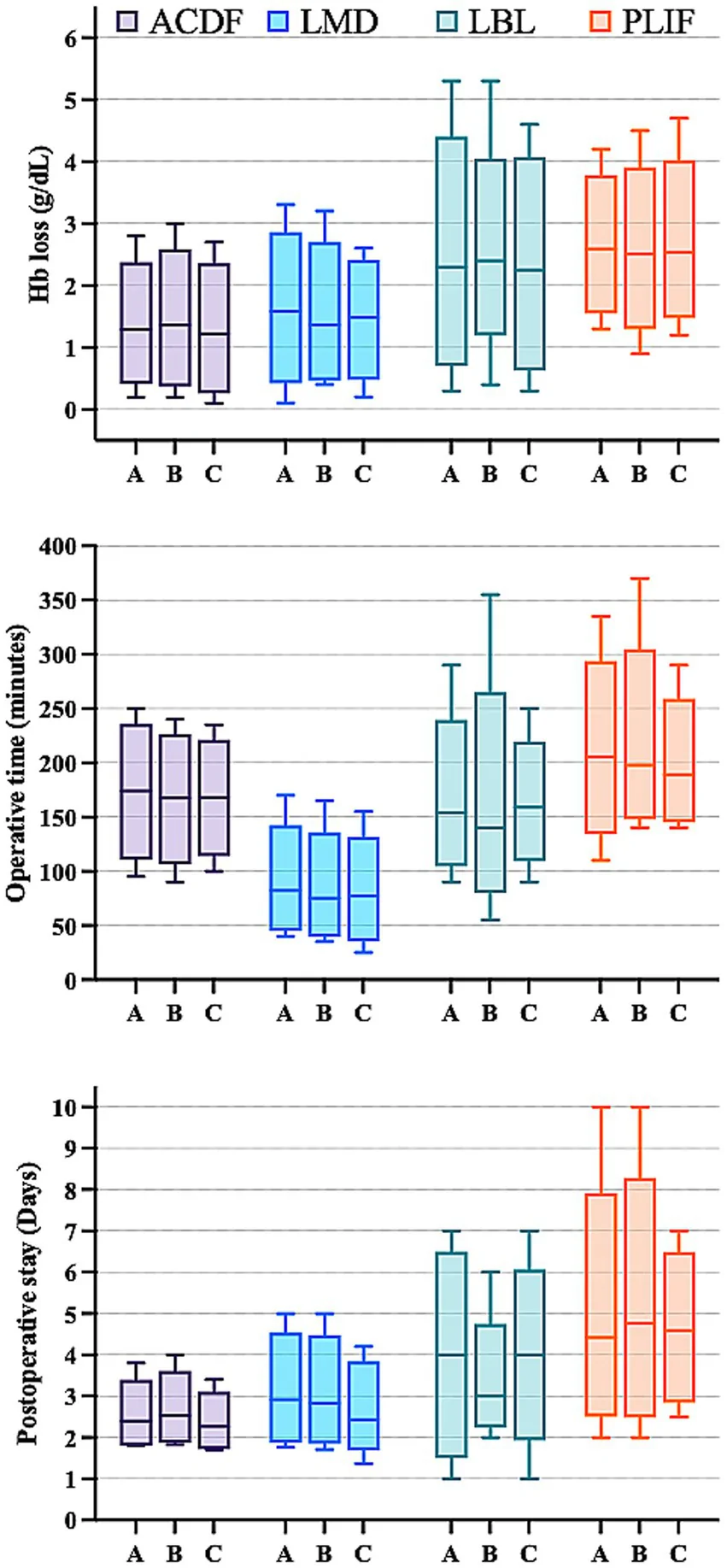
Mean ± SD plots showing perioperative outcomes across the four surgical procedures: anterior cervical discectomy and fusion (ACDF), lumbar microdiscectomy (LMD), lumbar laminectomy (LBL), and posterior lumbar interbody fusion (PLIF). For each procedure, outcomes were stratified according to the three antithrombotic management groups (A, B, and C). The upper panel illustrated intraoperative hemoglobin loss (g/dL), the middle panel operative time (minutes), and the lower panel postoperative length of stay (days). Error bars represent the mean ± standard deviation, and whiskers indicate the minimum and maximum values.

Intraoperative hemoglobin loss varied according to procedure type but did not differ across antithrombotic groups. In ACDF, mean losses were 1.29 ± 0.66 g/dL in Group A, 1.36 ± 0.81 g/dL in Group B, and 1.22 ± 0.80 g/dL in Group C. In LDH, values were 1.58 ± 0.84 g/dL, 1.36 ± 0.83 g/dL, and 1.48 ± 0.73 g/dL in Groups A, B, and C, respectively. In LSS, mean hemoglobin loss averaged 2.27 ± 1.26 g/dL across the cohort, with overlapping values among all groups. In LSL, mean losses were 2.54 ± 0.79 g/dL across groups, without significant differences (Figure 2).

Operative time remained consistent among the three antithrombotic groups within each procedure. In ACDF, In ACDF, operative time was 174.2 ± 47.7 min in Group A, 167.6 ± 45.1 min in Group B, and 168.0 ± 39.7 min in Group C. In LDH, operations lasted 82.4 ± 32.4, 75.1 ± 31.0, and 77.0 ± 31.3 min in Groups A, B, and C, respectively. In LSS, operative time was 154 ± 35 min in Group A, 140 ± 35 min in Group B, and 159 ± 30 min in Group C. In LSL, monosegmental PLIF procedures lasted 205.6 ± 46.9 min, 197.9 ± 42.0 min, and 189.2 ± 39.0 min in Groups A, B, and C.

Drain output, measured in LSS and LSL, remained consistent across groups. In LSS, drainage volumes averaged 170 ± 32 mL in all three groups. In LSL, mean drainage was 155 ± 33 mL, again without significant intergroup variation.

### 3.3 Postoperative course

In ACDF, length of stay ranged from 2.27 to 2.53 days among the groups. In LDH, hospitalization ranged from 2.43 to 2.92 days, with Group C showing a statistically shorter stay compared to Group A. In LSS, mean hospitalization was 3.58 days, and Group B had a significantly shorter stay than Group A. In LSL, length of stay averaged 4.6 days, with no significant differences across groups.

The observed between-group differences for hemoglobin loss, operative time, and postoperative hospitalization remained within the predefined clinically meaningful margins.

### 3.4 Functional outcomes

In ACDF, m-JOA increased from a mean of 14.7 to values between 16.5 and 16.8, while ODI decreased from 44.5 to between 24 and 27. In LDH, VAS improved from 7.3 to values between 2.4 and 2.6. In LSS, ODI decreased from approximately 41 to 34 points, and in LSL from approximately 53 to 38, with no significant intergroup differences.

### 3.5 Complications

In ACDF, all events were Clavien–Dindo grade I or II, with no reoperations for hematoma. LDH procedures showed a similar profile, with grade I and II complications only. In LSS, grade I complications were the most frequent, followed by grade II events and four grade III complications due to dural tears requiring repair. In LSL, complications were primarily grade I, with a limited number of grade II events and four grade III complications related to wound or implant issues. Across all procedures, no cases of postoperative epidural hematoma requiring evacuation, myocardial infarction, stroke, deep vein thrombosis, or pulmonary embolism were observed, and complication rates did not differ significantly among groups. No patient required intraoperative or postoperative blood transfusion.

Intraoperative outcomes, postoperative course, functional outcomes, and complication rates are summarized in Table 2.

**Table 2:** Perioperative and clinical outcomes according to surgical procedure and perioperative antithrombotic management strategy.

Model	All patients	Group A*	Group B^†^	Group C^‡^	Statistics
ACDF	189	69 (37)	72 (38)	48 (25)	
INR	1.05 (±0.12)	1.04 (±0.12)	1.06 (±0.13)	1.03 (±0.11)	*p* = 0.48
Preop m-JOA	14.7 (±1.9)	14.3 (±2.1)	14.7 (±1.8)	15.1 (±1.6)	*p* = 0.49
3-month m-JOA	16.5 (±1.5)	16.2 (±1.6)	16.5 (±1.5)	16.8 (±1.3)	*p* = 0.32
Preop ODI	44.5 (±11.5)	44.5 (±11.6)	45.8 (±12.2)	42.6 (±10.5)	p = 0.77
3-month ODI	25.6 (±8.2)	27.1 (±8.9)	25.3 (±8.4)	24.0 (±7.2)	*p* = 0.55
Clavien–Dindo score					
Grade I	22 (12)	7 (10)	8 (11)	7 (15)	p = 0.8
Grade II	9 (5)	4 (6)	2 (3)	3 (6)	
Grade III	0	0	0	0	
Operative hemoglobin loss, g/dL	1.3 (±0.76)	1.29 (±0.66)	1.36 (±0.81)	1.22 (±0.80)	A vs. B p = 0.77; A vs. C p = 0.82
Median (IQR)	1.39 (0.79–1.69)	1.30 (0.85–1.71)	1.40 (0.78–1.90)	1.27 (0.67–1.87)	
Operative time, min	170.1 (±44.64)	174.2 (±47.69)	167.6 (±45.1)	168.0 (±39.71)	A vs. B *p* = 0.47; A vs. C *p* = 0.52
Median (IQR)	162 (138–197)	170 (135–210)	173 (140–206)	165 (143–194)	
Postoperative hospitalization, days	2.42 (±0.63)	2.40 (±0.59)	2.53 (±0.70)	2.27 (±0.54)	A vs. B *p* = 0.45; A vs. C p = 0.82
Median (IQR)	2 (2–3)	2 (2–3)	3 (2–3)	2 (2–3)	
LMD	163	63 (39)	54 (33)	46 (28)	
INR	1.05 (±0.53)	1.04 (±0.51)	1.07 (±0.56)	1.05 (±0.52)	p = 0.48
Preop VAS	7.3 (±2.4)	7.3 (±2.5)	7.2 (±2.4)	7.4 (±2.3)	*p* = 0.81
3-month VAS	2.5 (±1.2)	2.5 (±0.9)	2.6 (±1.3)	2.4 (±1.4)	*p* = 0.93
Clavien–Dindo score					
Grade I	23 (14)	6 (10)	8 (15)	9 (20)	p = 0.45
Grade II	8 (5)	3 (5)	3 (6)	2 (4)	
Grade III	0	0	0	0	
Operative hemoglobin loss, g/dL	1.48 (±0.81)	1.58 (±0.84)	1.36 (±0.83)	1.48 (±0.73)	A vs. B *p* = 0.58; A vs. C p = 0.20
Median (IQR)	1.37 (0.90–1.89)	1.48 (0.82–1.80)	1.34 (0.76–1.92)	1.41 (0.99–1.87)	
Operative time, min	78.46 (±31.63)	82.37 (±32.41)	75.11 (±31.04)	77.04 (±31.35)	A vs. B *p* = 0.46; A vs. C *p* = 0.19
Median (IQR)	82 (62–103)	83 (60–105)	78 (58–96)	76 (60–107)	
Postoperative hospitalization, days	2.75 (±1.14)	2.92 (±1.16)	2.83 (±1.12)	2.43 (±1.06)	A vs. B *p* < 0.01; A vs. C *p* = 0.68
Median (IQR)	3 (2–3)	3 (2–4)	3 (2–4)	2 (2–3)	
LBL	226	95 (42)	43 (19)	88 (39)	
INR	1.01 (±0.05)	1.02 (±0.07)	1.01 (±0.08)	1.01 (±0.08)	*p* = 0.42
Preop ODI	41.22 (±16.54)	40.75 (±17.74)	43.61 (±18.32)	41.72 (±16.25)	*p* = 0.24
3-month ODI	34.23 (±18.35)	33.50 (±14.78)	35.85 (±16.52)	35.47 (±15.04)	*p* = 0.12
Clavien–Dindo score					
Grade I	51 (23)	23 (24)	10 (23)	18 (21)	p = 0.67
Grade II	11 (5)	5 (5)	2 (5)	4 (4)	
Grade III	4 (2)	2 (2)	1 (2)	1 (1)	
Drain fluid output (mL)	170 (±65)	175 (±60)	165 (±60)	165 (±65)	
Operative hemoglobin loss, g/dL	2.27 (±1.26)	2.3 (±1.2)	2.4 (±0.4)	2.25 (±1.3)	A vs. B *p* = 0.76; A vs. C p = 0.58
Median (IQR)	2.20 (1.38–3.02)	2.26 (1.41–3.14)	2.36 (2.02–2.67)	2.16 (1.45–3.0)	
Operative time, min	155 (±37)	154 (±35)	140 (±35)	159 (±30)	A vs. B p = 0.15; A vs. C *p* = 0.87
Median (IQR)	152 (125–183)	157 (133–182)	143 (127–171)	156 (131–176)	
Postoperative hospitalization, days	3.58 (±2.08)	4 (±2)	3 (±0.5)	4 (±1.14)	A vs. B *p* = 0.01; A vs. C *p* = 0.71
Median (IQR)	4 (2–5)	4 (3–5)	3 (2–4)	4 (3–5)	
PLIF	189	74 (39)	87 (46)	28 (15)	
INR	0.91 (±0.09)	0.91 (±0.06)	0.91 (±0.11)	0.93 (±0.07)	p = 0.12
Preop ODI	52.61 (±17.35)	50.43 (±14.87)	55.12 (±18.03)	52.29 (±19.87)	p = 0.32
3-month ODI	37.81 (±14.29)	35.42 (±11.89)	37.23 (±13.76)	40.78 (±17.92)	p = 0.17
Clavien–Dindo score					
Grade I	45 (24)	15 (20)	22 (25)	8 (29)	p = 0.71
Grade II	14 (7)	6 (8)	6 (7)	2 (7)	
Grade III	4 (2)	1 (1)	2 (2)	1 (4)	
Drain fluid output (mL)	155 (±33)	145 (±28)	165 (±38)	155 (±32)	*p* = 0.23
Operative hemoglobin loss, g/dL	2.54 (±0.79)	2.59 (±0.76)	2.51 (±0.81)	2.54 (±0.79)	A vs. B *p* = 0.56; A vs. C *p* = 0.79
Median (IQR)	2.49 (1.95–3.02)	2.53 (2.03–3.10)	2.47 (1.89–2.95)	2.48 (1.96–3.07)	
Operative time, min	199.6 (±43.71)	205.6 (±46.9)	197.9 (±41.99)	189.2 (±38.95)	A vs. B *p* = 0.27; A vs. C *p* = 0.08
Median (IQR)	205 (167–230)	210 (175–243)	200 (171–233)	193 (161–218)	
Postoperative hospitalization, days	4.6 (±1.6)	4.42 (±1.4)	4.77 (±1.8)	4.59 (±1.4)	A vs. B p = 0.19; A vs. C *p* = 0.60
Median (IQR)	5 (4–7)	5 (3–6)	5 (4–7)	5 (4–6)	

Values are reported as mean ± standard deviation or number (%). Continuous variables were compared using the Kruskal–Wallis test, whereas categorical variables were analyzed using the Chi-square test. Pairwise comparisons were performed using the Mann–Whitney U test, when appropriate. ACDF = anterior cervical discectomy and fusion; LMD = lumbar microdiscectomy; LBL = bilateral lumbar laminectomy for spinal stenosis; PLIF = posterior lumbar interbody fusion; ODI = Oswestry Disability Index; VAS = Visual Analog Scale; m-JOA = modified Japanese Orthopaedic Association score; INR = international normalized ratio.

*Group A: patients not receiving chronic antiplatelet (AP) or anticoagulant (AC) therapy.

^†^Group B: patients receiving AP or AC therapy for primary prophylaxis who underwent perioperative discontinuation and substitution with low-molecular-weight heparin (LMWH).

^‡^Group C: patients receiving AP or AC therapy for secondary prophylaxis who underwent temporary discontinuation and perioperative management with low-dose aspirin.

## 4 Discussion

### 4.1 Establishing a niche

Degenerative spine disease represents one of the most common indications for elective spinal surgery in the elderly population, a demographic characterized by a high prevalence of cardiovascular and cerebrovascular comorbidities requiring chronic antithrombotic therapy (36, 42, 43). As life expectancy continues to increase, a growing proportion of patients undergoing cervical and lumbar procedures receive anticoagulant or antiplatelet medications at the time of surgical evaluation (23, 25, 44). Consequently, perioperative management in this setting requires balancing two competing priorities: minimizing hemorrhagic complications associated with spinal surgery while maintaining adequate protection against venous thromboembolism (VTE) and major cardio-cerebrovascular events (45). This clinical scenario has progressively become a relevant challenge in contemporary spine practice.

Symptomatic postoperative spinal epidural hematoma (SEH), although relatively infrequent, remains one of the most feared complications after spinal surgery because of its potential to cause severe neurological deterioration (11, 13, 46). Large meta-analyses have estimated an overall incidence of approximately 0.5%, with higher rates reported following posterior decompressive procedures (47). Several perioperative factors have been associated with an increased risk of SEH, including lumbar surgery, prolonged operative time, hypertension, and greater intraoperative blood loss (48).

Beyond the risk of clinically significant SEH, the presence of ongoing antithrombotic therapy is also commonly perceived as a potential contributor to increased intraoperative bleeding, which may complicate surgical exposure and raise concerns regarding postoperative hematoma formation (48). However, available evidence suggests that the development of SEH is multifactorial and may not be predominantly driven by antithrombotic exposure alone (5, 40, 49). Overall, these observations indicate that perioperative bleeding risk in spine surgery results from a complex interaction between patient-related factors, procedural characteristics, and pharmacological therapy (7, 30, 45, 50).

Thromboembolic complications represent the opposite side of the perioperative risk spectrum in patients undergoing spine surgery while receiving antithrombotic therapy. Deep venous thrombosis and pulmonary embolism remain clinically relevant concerns in this population, particularly among elderly patients with cardiovascular comorbidities and reduced postoperative mobility. Episodes of deep venous thrombosis and occasional postoperative hematoma have been reported despite the use of prophylactic strategies, highlighting the narrow therapeutic margin between effective thromboprophylaxis and bleeding safety (51).

### 4.2 Interpretation of results

The present multicenter retrospective cohort analysis evaluated perioperative antithrombotic management across four common degenerative spine procedures, ACDF, LMD, LBL, and PLIF, in a cohort of 767 consecutive patients. Perioperative outcomes, including intraoperative blood loss, operative time, postoperative hospitalization, and complications, were compared among patients undergoing LMWH substitution, continuation of low-dose aspirin, and those not receiving chronic antithrombotic therapy.

This analysis provides an integrated assessment of perioperative outcomes associated with different antithrombotic management strategies across a heterogeneous spectrum of degenerative spinal procedures.

Across all procedures, perioperative parameters remained largely comparable among groups, and no major hemorrhagic or thromboembolic events were observed. Intraoperative blood loss and operative duration did not significantly differ among the different antithrombotic management strategies, suggesting that both LMWH substitution and temporary continuation of low-dose aspirin did not result in clinically relevant perioperative bleeding in these procedures. These findings were consistent with previous reports indicating that structured perioperative antithrombotic protocols may be safely applied in elective spinal surgery when meticulous surgical hemostasis and standardized perioperative pathways are adopted. Our findings are consistent with recent reports showing comparable safety profiles between LMWH and factor Xa inhibitors in both degenerative spine surgery and nonoperative spinal trauma, although these studies investigated postoperative thromboprophylaxis rather than perioperative management of chronic antithrombotic therapy (52).

Importantly, the consecutive nature of the cohort and the overall homogeneity of baseline clinical and surgical characteristics across groups strengthened the internal consistency of the analysis (7, 31). Demographic variables, comorbidity profiles, and procedure-specific characteristics were comparable among treatment groups, reducing the likelihood that the observed perioperative outcomes were influenced by relevant baseline imbalances. In this context, the absence of significant differences in the primary perioperative variables appeared to reflect genuine procedural comparability rather than confounding effects related to patient selection.

Overall, the absence of increased bleeding or complication rates in patients undergoing substitution-based antithrombotic strategies suggested that perioperative cardiovascular protection could be maintained without substantially compromising surgical safety in elective degenerative spine procedures.

These findings align with the growing body of literature suggesting that carefully structured perioperative antithrombotic protocols may allow safe continuation or substitution strategies in selected spinal procedures.

### 4.3 Updated guidelines and recommendations

Current evidence addressing this balance in degenerative spine surgery remains limited. Systematic reviews have emphasized the scarcity of high-quality, procedure-specific data capable of guiding perioperative chemoprophylaxis while adequately accounting for hemorrhagic complications (16). Consequently, perioperative antithrombotic management in degenerative spine procedures continues to rely largely on heterogeneous evidence and institutional practice patterns rather than on robust procedure-specific data (51, 53, 54).

Several clinical guidelines have addressed perioperative antithrombotic management in patients undergoing spinal procedures; however, procedure-specific recommendations remain limited. The evidence-based guideline issued by the North American Spine Society represents a cornerstone reference for spine surgeons but provides only limited stratification according to individual degenerative procedures (5).

Similarly, recommendations developed by the American Society of Regional Anesthesia and Pain Medicine and collaborating societies were primarily intended for neuraxial and interventional pain procedures and therefore offer only indirect guidance for elective spine surgery (7).

More recent perioperative recommendations addressing anticoagulants and direct oral anticoagulants have categorized spine surgery within the broader group of high–bleeding-risk procedures, yet they do not differentiate among the heterogeneous spectrum of degenerative spinal operations (55, 56).

The absence of uniform guidance is reflected in considerable variability in real-world clinical practice. Surveys among spine surgeons have consistently demonstrated substantial heterogeneity in perioperative antithrombotic management, often driven by institutional practice patterns rather than standardized recommendations (57).

International survey data from the AO Spine Anticoagulation Global Survey further documented marked discrepancies in anticoagulant interruption intervals, bridging strategies, and postoperative resumption across different regions and healthcare systems (45). Similar variability has also been reported for the perioperative management of direct oral anticoagulants, particularly factor Xa inhibitors, reflecting the absence of broadly accepted procedural guidelines in degenerative spine surgery (58).

The evidence supporting perioperative continuation of LDA in spine surgery has progressively strengthened over the past decade. Systematic reviews and meta-analyses have consistently shown that aspirin continuation does not significantly increase intraoperative blood loss, operative duration, or overall perioperative complication rates in patients undergoing spinal procedures (10).

More recent retrospective series have further confirmed the safety of maintaining low-dose aspirin during lumbar decompression and minimally invasive posterior procedures, with no significant differences observed in transfusion requirements, reoperation for postoperative hematoma, or overall surgical complication rates (12, 59).

In contrast to the growing body of evidence regarding aspirin continuation, data addressing the perioperative management of direct oral anticoagulants (DOACs) in spine surgery remain limited. Available clinical observations suggest that shortened interruption intervals may be compatible with acceptable perioperative bleeding profiles; however, the current evidence base is constrained by small cohorts, procedural heterogeneity, and variability in postoperative resumption strategies (60).

Systematic evaluations have similarly highlighted the persistent uncertainty surrounding the role and timing of chemoprophylaxis in degenerative spine procedures, particularly in relation to balancing thromboembolic prevention with hemorrhagic safety (16). Collectively, these findings indicate that perioperative antithrombotic management in degenerative spine surgery remains incompletely defined, with limited procedure-specific evidence available to guide clinical decision-making.

Overall, current guidelines and available studies provide an important conceptual framework but do not fully address several practical aspects of perioperative antithrombotic management in degenerative spine surgery. In particular, robust multicenter evidence comparing LMWH substitution strategies and continuation of low-dose aspirin with patients not receiving chronic antithrombotic therapy remains limited.

Given the increasing proportion of elderly patients undergoing spinal procedures while receiving long-term antithrombotic treatment, together with the substantial heterogeneity of perioperative practices across institutions, further large-cohort clinical evidence is required to better characterize the safety profile of different management strategies and to inform more consistent perioperative decision-making.

### 4.4 Clinical implications

The present findings suggested that structured perioperative antithrombotic management may be compatible with acceptable surgical safety in common degenerative spine procedures. Across cervical and lumbar operations, perioperative strategies based on LMWH substitution or continuation of low-dose aspirin did not appear to result in clinically relevant increases in intraoperative blood loss, operative time, postoperative hospitalization, or complication rates when compared with patients not receiving chronic antithrombotic therapy.

These observations were consistent with growing evidence indicating that continuation of low-dose aspirin during spinal procedures may not significantly increase perioperative bleeding or operative complexity in selected surgical settings (10, 11). At the same time, interruption of antithrombotic therapy has been associated with a measurable risk of thromboembolic complications in patients with underlying cardiovascular disease, highlighting the importance of balancing hemorrhagic and thrombotic risks during the perioperative period (3).

Within this context, the present multicenter experience suggested that protocol-driven perioperative strategies, such as LMWH substitution or continuation of low-dose aspirin, may represent a reasonable approach in degenerative spine surgery when combined with careful patient selection and meticulous surgical hemostasis. These findings also aligned with previous surveys showing substantial heterogeneity in perioperative antithrombotic management among spine surgeons, underlining the potential value of more standardized clinical pathways (45).

### 4.5 Limitations

Several limitations should be acknowledged when interpreting the findings of the present study.

First, the retrospective observational design inherently exposed the analysis to potential sources of selection bias and unmeasured confounding. Accordingly, no *a priori* sample size calculation was performed, as the study was designed to include all consecutive eligible patients treated during the predefined study period rather than a predetermined recruitment target. Although the inclusion of a consecutive multicenter cohort and the overall homogeneity of baseline characteristics across groups partially mitigated selection bias, the present study was not specifically designed to evaluate very rare thromboembolic or hemorrhagic complications. Therefore, the absence of such events should be interpreted with appropriate caution and should not be considered definitive evidence of their absence.

Second, the study was conducted across multiple centers, which may have introduced variability in perioperative management, surgical technique, and postoperative care. Nevertheless, the use of a standardized antithrombotic protocol across institutions aimed to reduce this potential heterogeneity. Although no postoperative epidural hematoma requiring surgical evacuation or major thromboembolic events were observed, the present study was not specifically designed to detect very rare complications. Moreover, asymptomatic thromboembolic events were not actively screened through routine postoperative imaging. Therefore, the absence of these events should be interpreted with appropriate caution and should not be considered definitive evidence regarding the safety of the evaluated perioperative antithrombotic strategies. Hemoglobin loss was estimated from the difference between the last preoperative and the first postoperative hemoglobin values and therefore did not account for the potential influence of intraoperative blood transfusions on postoperative hemoglobin levels.

Another limitation was the absence of detailed pharmacological stratification among different classes of anticoagulant agents, particularly direct oral anticoagulants, which may exhibit distinct pharmacokinetic profiles and perioperative management considerations. In addition, the observational design did not allow formal causal inference regarding the relationship between antithrombotic strategy and perioperative outcomes. The comparison among the three study groups may have been influenced by confounding by indication, as perioperative antithrombotic management was determined by the underlying cardiovascular risk profile rather than by random allocation. Furthermore, the present findings should not be extrapolated to patients receiving VKA or dual antiplatelet therapy, as the bleeding risk of these patients required dedicated perioperative management strategies. Therefore, these patients were considered not eligible, and not represented in the study cohort. Detailed information regarding the specific preoperative antithrombotic regimen and its underlying clinical indication was not systematically available for all patients and therefore could not be evaluated. Moreover, although ODI was reported in the ACDF cohort, it should be interpreted as a complementary measure of overall disability rather than a cervical-specific functional outcome. Although all participating centers adopted a shared study protocol, potential center-specific effects could not be evaluated and therefore cannot be completely excluded. No adjustment for multiple comparisons was performed, which may have increased the risk of type I error.

Finally, although the cohort represented one of the largest multicenter series evaluating perioperative antithrombotic management across multiple degenerative spine procedures, the results should be interpreted within the context of elective surgery performed in experienced centers and may therefore not be fully generalizable to all clinical settings.

## 5 Conclusion

In this multicenter retrospective cohort of 767 consecutive patients undergoing common degenerative spine procedures, perioperative antithrombotic management strategies based on LMWH substitution or continuation of LDA were not associated with clinically relevant increases in intraoperative blood loss, operative time, postoperative hospitalization, or complication rates when compared with patients not receiving chronic antithrombotic therapy.

Within the limitations of this retrospective multicenter study, standardized perioperative LMWH substitution and temporary perioperative substitution with low-dose aspirin were not associated with an increased incidence of major perioperative complications. However, these findings should not be interpreted as definitive evidence of safety for rare thromboembolic or hemorrhagic events.

The consecutive nature of the cohort, the homogeneity of baseline clinical characteristics, and the results of our study strengthened the interpretation that structured perioperative antithrombotic pathways may be compatible with acceptable surgical safety in degenerative spine surgery.

Taken together, these findings suggested that protocol-driven perioperative antithrombotic management may represent a reasonable strategy for balancing hemorrhagic and thromboembolic risks in elderly patients undergoing elective cervical and lumbar spinal procedures.

## Data Availability

The raw data supporting the conclusions of this article will be made available by the authors, without undue reservation.
